# SLC2A1 and MPST as diagnostic and prognostic biomarkers of potential endometrial cancer

**DOI:** 10.3389/fimmu.2025.1575916

**Published:** 2025-07-17

**Authors:** Xiaoyu Xi, Xinxin Gong, Yixi Liu, Boran Cui, Chenchen Xia, Shan Qin, Jiexian Du

**Affiliations:** ^1^ Department of Gynecology, The Second Hospital of Hebei Medical University, Shijiazhuang, Hebei, China; ^2^ Clinical College of Hebei Medical University, Shijiazhuang, Hebei, China

**Keywords:** SLC2A1, MPST, UCEC, clinical outcome, immune cell infiltration, immune checkpoint

## Abstract

Uterine corpus endometrial carcinoma (UCEC) is the predominant neoplasm affecting the female reproductive system. Early diagnosis of UCEC is crucial for improving patient survival rates. In this study, we selected and investigated two specific genes associated with hydrogen sulfide(H_2_S): SLC2A1, which encodes a glucose transporter, and MPST, which encodes 3-mercaptopyruvate thiotransferase. Both SLC2A1 and MPST have been identified as important regulators in cancer. The objective of this study was to investigate the potential significance of SLC2A1 and MPST in terms of UCEC diagnosis and prognosis. Our analysis using Kaplan-Meier survival curves and receiver operating characteristic (ROC) curves demonstrated robust diagnostic and prognostic significance for both SLC2A1 and MPST. Moreover, our research revealed a significant association between the expression levels of SLC2A1 and MPST, immune cell infiltration, immune checkpoint gene presence, and TP53 in UCEC tissues. Furthermore, we observed that DNA methylation status of the CpG island of SLC2A1 and the MPST gene is associated with UCEC prognosis. These findings suggest that SLC2A1 and MPST genes hold promise in distinguishing endometrial cancer patients from normal cases, highlighting their diagnostic and prognostic potential as biomarkers for UCEC. These results offer encouraging prospects for targeted therapies.

## Introduction

The incidence of uterine corpus endometrial carcinoma (UCEC), a prevalent form of gynecological cancer, is steadily increasing worldwide (1). In recent years, the rising prevalence of obesity, lifestyle changes, and the increased use of estrogen replacement therapy have significantly contributed to the surge in UCEC cases, posing a substantial health risk to women (2). Currently, surgery remains the primary treatment option for UCEC; however, it is crucial to select appropriate adjuvant therapies based on the tumor’s pathology and clinical stage. Therefore, identifying suitable gene targets for UCEC treatment and establishing reliable diagnostic and prognostic indicators are essential. These advancements present an opportunity to explore novel immunotherapy strategies. Following nitric oxide and carbon monoxide, hydrogen sulfide (H_2_S) has been recognized as the third gas signaling molecule and is found abundantly in mammals. Endogenous H_2_S is primarily produced by enzymes such as cystathionine β-synthase (CBS), cystathionine lyase (CSE), and 3-mercaptopyruvate sulfurtransferase (3-MST) (3). The coding gene for cystathionine gamma-lyase (CSE) is CTH, 3-MST is encoded by the MPST gene. CBS, in contrast, is encoded by the cystathionine β-synthase gene.

Fluctuations in cystathionine β-synthase (CBS) expression are associated with alterations in hydrogen sulfide (H_2_S) levels, contributing to the development of pathological conditions in various biological systems, including the brain, heart tissue, immune system, and liver. Under physiological conditions, the liver and brain are the primary sites of CBS expression. Extensive research has highlighted the significance of H_2_S in obstetrical and gynecological diseases, particularly in conditions such as endometriosis (3) and gestational hypertension (4, 5). These studies emphasize the crucial role of H_2_S in mitigating inflammation, thereby aiding in the management of these gynecological ailments. However, our investigation of endometrial cancer revealed minimal expression of CBS.

As a facilitative glucose transporter, SLC2A1 is responsible for the continuous, or basal, uptake and transport of glucose (6). The glucose transporter proteins (GLUTs) are crucial membrane proteins that mediate the transmembrane transport of glucose, maintaining cellular energy supply and supporting normal cellular functions.​

The overexpression of SLC2A1 in various types of cancer, including breast, lung, liver, endometrial, oral, Colorectal and gastric cancers, is particularly intriguing (7–14). For instance, studies have shown that in breast cancer, SLC2A1 overexpression is associated with upregulation of genes involved in the PI3K - AKT - mTOR pathway, which promotes cell proliferation and survival (15). In lung cancer, SLC2A1 has been found to interact with KRAS, another key cancer gene, enhancing the Warburg effect and tumorigenesis (16). Nevertheless, the role of SLC2A1 in uterine corpus endometrial carcinoma (UCEC) remains not fully understood.

The coding gene for 3-mercaptopyruvate sulfurtransferase (3-MST) is MPST, which is a key enzyme regulating the biosynthesis of endogenous hydrogen sulfide (H2S) and is expressed in various human tissues. The activation of MPST is involved in important processes such as tRNA sulfuration, protein aminoacylation, and cyanide detoxification (4). Experimental results indicate that MPST plays a significant role in providing protection against oxidative stress, overseeing mitochondrial function in respiration, and managing fatty acid metabolism (4). MPST is expressed in a variety of cancer tissues, such as glioblastoma, lung cancer, colon cancer, head and neck squamous cell carcinoma, liver cancer, pancreatic cancer, renal cell carcinoma, etc., and the expression level and prognostic significance of MPST are different in different cancers, suggesting that MPST may be a cancer-related gene (17). Furthermore, research has shown that MPST is present in endometrial tumors, indicating a direct connection to the survival rate of patients diagnosed with uterine corpus endometrial carcinoma. However, there remains a gap in understanding the specific functions of MPST in this context, including its clinical correlation analysis and functional pathway enrichment analysis, which have not been extensively investigated.

In this study, we investigate the expression of SLC2A1 and MPST across various types of cancer. However, the specific roles of SLC2A1 and MPST in tumor immune cell infiltration, abnormal DNA methylation, and prognosis in uterine corpus endometrial carcinoma (UCEC) have not yet been elucidated. Therefore, through comprehensive bioinformatics analysis of the TCGA database, our study aims to provide a visual representation of the diagnostic and prognostic significance of SLC2A1 and MPST in UCEC. Furthermore, we conducted an interrelated analysis of SLC2A1 and MPST methylation and examined whether gene alterations impact disease outcomes in patients. Additionally, we validated the differential expression of SLC2A1 and MPST in UCEC and normal tissues through experiments, confirming the beneficial roles of SLC2A1 and MPST in the diagnosis and treatment of UCEC patients, suggesting that they may serve as therapeutic targets for the development of novel immunotherapy strategies.

## Materials and methods

### Source and treatment of the samples

We obtained RNA sequencing (RNAseq) data along with relevant clinical information for 587 samples from the TCGA-UCEC project of The Cancer Genome Atlas (https://portal.gdc.cancer.gov/). After excluding samples that lacked clinical information and removing duplicates, we converted the RNA sequencing data from the FPKM (Fragments Per Kilobase of transcript per Million mapped reads) format to the TPM (Transcripts Per Million) format. Based on the median expression values of SLC2A1 and MPST, we categorized UCEC patients into low and high expression groups. The statistical analysis was visualized using the ggplot2 software package and performed with R software version 3.6.3. The Wilcoxon rank-sum test revealed two datasets that were statistically significant (*P* < 0.05). We defined statistical significance as follows: *** (*P* < 0.001), ** (*P* < 0.01), * (*P* < 0.05), and ns (*P* > 0.05). We confirm that all necessary informed consent was obtained prior to data collection, in accordance with the guidelines for accessing the publicly available TCGA database.

The present study received approval from the Ethics Committee of the Second Hospital of Hebei Medical University (Hebei, China). All patients were informed about the study and provided their consent to participate. Paraffin-embedded specimens were collected from 27 patients diagnosed with Uterine Corpus Endometrial Carcinoma (UCEC) and 27 normal control subjects at the Second Hospital of Hebei Medical University.

### Clinical correlation analysis

We conducted a correlation analysis of SLC2A1 and MPST with tumor stage using R software, specifically utilizing the ggplot2 package. The variables considered in the analysis included clinical stage, histological grade, pathological stage, and histological type. Furthermore, we generated a Kaplan-Meier plot and performed diagnostic ROC curve analysis using the R packages pROC and ggplot2.

### Construction of the protein-protein interaction network and GO-KEGG analysis

To visualize the protein-protein interaction (PPI) network, we utilized Cytoscape (version 3.7.2) and accessed the STRING database (https://string-db.org/). A protein interaction score threshold of 0.4 was established to determine statistically significant interactions. Subsequently, we identified 10 functional partner genes for further analysis, focusing on Gene Ontology (GO) term enrichment and KEGG pathway analysis to gain insights into the functions of SLC2A1 and MPST.

### Methylation

We investigated the DNA methylation levels of the SLC2A1 and MPST genes and assessed the prognostic significance of the CpG islands within these genes for patients with Uterine Corpus Endometrial Carcinoma (UCEC). This analysis was conducted using the MetSurv database (https://biit.cs.ut.ee/methsurv/). Statistical significance was defined as a P-value of less than 0.05.

### Analysis of the immune infiltration

We utilized the study by Bindea et al. (18) to extract marker genes for 24 immune cell types. Using Gene Set Enrichment Analysis (GSEA) with single-sample GSEA (ssGSEA) on UCEC mRNA TPM data, we calculated the levels of tumor-infiltrating immune cells (19). We performed a correlation analysis between the expression levels of the SLC2A1 and MPST genes, as well as the relationships between immune checkpoint genes (including CD96, CTLA4, and PDCD1) and TP53. This analysis was conducted on UCEC samples from the TCGA database using Spearman’s correlation analysis and the “ggplot2” (v3.3.3) R package. Correlations were considered significant if the P-value was below 0.05.

### Gene alterations in UCEC samples

We utilized cBioPortal (https://www.cbioportal.org/) to conduct log-rank tests and survival curve analysis. These analyses were performed to assess the prognostic significance of genomic alterations in the SLC2A1 and MPST genes. Statistical significance was determined using a threshold of *P* < 0.05.

### Assessment of the prognostic significance of MPST expression in UCEC

To evaluate the survival outcomes of UCEC patients, we conducted Kaplan-Meier survival curve analysis and both multivariate and univariate Cox regression analyses, focusing on the expression levels of SLC2A1 and MPST. We divided TCGA samples into high and low expression groups based on the median expression levels of SLC2A1 and MPST genes in UCEC. Furthermore, we employed the “pROC” (v1.17.0.1), “timeROC” (v0.4), and “ggplot2” (v3.3.3) R packages to perform diagnostic ROC curve and nomogram model analyses. The purpose of these analyses was to determine whether MPST expression levels can be used to predict UCEC diagnosis.

### Immunohistochemistry

IHC staining was performed according to the outlined protocol. In brief, the slides were dewaxed and dehydrated, followed by thermal-induced antigen retrieval using SLC2A1 (item number: GB11215) and MPST (item number: 19499-1-AP) in PBS (pH 7.4). A 3% hydrogen peroxide solution was used to block endogenous peroxidase activity, and 3% BSA was added dropwise to the tissue to ensure even coverage for serum blocking. The slides were incubated overnight with primary antibodies against SLC2A1 and MPST. After washing with PBS, the tissue was covered with a corresponding HRP-labeled secondary antibody and incubated at room temperature for 30 minutes. Following decolorization with PBS, a freshly prepared DAB colorimetric solution was added dropwise, with a positive result indicated by a brownish-yellow color. The slides were rinsed with tap water to terminate the colorimetric process, counterstained with hematoxylin, and returned to blue. They were then dehydrated through a series of ethanol washes, rinsed with running water, cleared with xylene, mounted, and observed under a microscope.

### qPCR

Total RNA was extracted from cells using Trizol reagent (Invitrogen). The quality of total RNA from the samples was assessed using Nanodrop and agarose gel electrophoresis. The Transcriptor First-Strand cDNA Synthesis Kit was used to synthesize complementary DNA. Quantitative PCR was performed using SYBR Green. For messenger RNA (mRNA) analysis, glyceraldehyde 3-phosphate dehydrogenase (GAPDH) served as the internal control. The relative expression levels of SLC2A1 and MPST were determined using relative quantitative methods. The primer sequences were as follows: H-SLC2A1-S: GCTTCTCCAACTGGACCTCAAA, H-SLC2A1-A: GAAGAACAGAACCAGGAGCACAG, H-MPST-S: CGCCTTCATCAAGACCTACGAG, and H-MPST-A: GGCTCAGGAAGTCTGTGAAGGG.

### Statistical analysis

For statistical analysis, we utilized R (v.3.6.3). Group differences were compared using either the Wilcoxon rank-sum test or the t-test. For the Kaplan-Meier survival analysis, we employed the log-rank test. In the Cox regression analysis, we calculated hazard ratios (HRs) and their corresponding 95% confidence intervals (CIs). In the regression analyses, we first performed univariate analyses of common clinical influencing factors to assess the association between each potential predictor and outcome. We then selected the most relevant variables for inclusion in the multivariable model on the basis of statistical significance (p < 0.1) in univariate analysis and on the basis of their theoretical relevance. Variables that were also statistically significant in the multivariate model were included in the prognostic model. At the same time,the Benjamini-Hochberg procedure was also applied to adjust the significance threshold.

## Results

### The expression levels of SLC2A1 and MPST genes were assessed in both normal tissues and tumor

**Figure 1:**
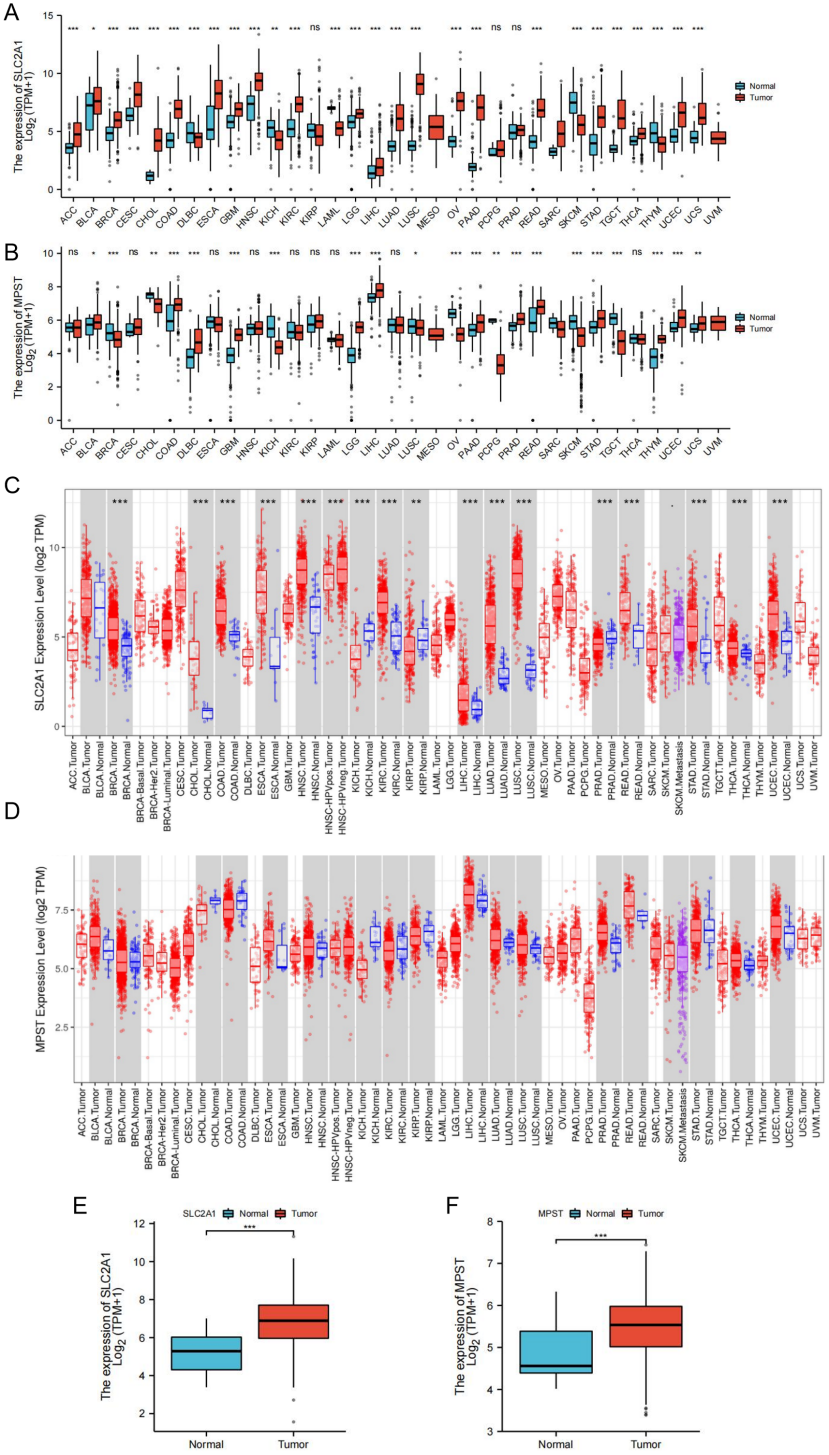
Expression levels of SLC2A1 and MPST genes in tumor and normal tissues. **(A)** The expression of SLC2A1 in TCGA tumors and normal tissues compared with GTEx database data; **(B)** The expression of MPST in TCGA tumors and normal tissues compared with GTEx database data; **(C, D)** Expressions of SLC2A1 and MPST in tumor grade normal tissues in TIMER database; **(E, F)** Expression level of SLC2A1 and MPST were significantly higher in UCEC tissue than in adjacent peritumor uterus tissue (*P<0.05, **P<0.01, ***P<0.001). ns stands for not statistically significant.

We analyzed the expression levels of the SLC2A1 and MPST genes using cancer datasets from the TCGA database. SLC2A1 was significantly upregulated in 22 out of 33 tumor tissues, covering diverse cancer types. These included adrenocortical cancer (ACC), bladder urothelial carcinoma (BLCA), breast invasive carcinoma (BRCA), cervical and endocervical cancer (CESC), cholangiocarcinoma (CHOL), colon adenocarcinoma (COAD), esophageal carcinoma (ESCA), glioblastoma multiforme (GBM), head and neck squamous cell carcinoma (HNSC), kidney clear cell carcinoma (KIRC), lower grade glioma (LGG), lung squamous cell carcinoma (LUSC), liver hepatocellular carcinoma (LIHC), lung adenocarcinoma (LUAD), ovarian serous cystadenocarcinoma (OV), pancreatic adenocarcinoma (PAAD), rectal adenocarcinoma (READ), stomach adenocarcinoma (STAD), testicular germ cell tumor (TGCT), thyroid carcinoma (THCA), uterine corpus endometrioid carcinoma (UCEC), and uterine carcinosarcoma (UCS) (**Figure 1A**).​

Likewise, MPST was upregulated in 13 of the 33 tumor tissues, namely in BLCA, COAD, DLBC, GBM, LGG, LIHC, PRAD, PAAD, READ, STAD, THYM, UCEC, and UCS (**Figure 1B**). Data from the TIMER database further corroborated these findings (**Figures 1C, D**). Significantly, both SLC2A1 and MPST were markedly upregulated in UCEC tissues (**Figures 1E, F**).

### Baseline data of the UCEC patients

In February 2023, we downloaded clinical and expression data for 543 UCEC cases from the TCGA database, as shown in **Table 1**. Among these cases, 206 patients (38.1% of the total cohort) were under 60 years old, and 343 patients (61.9%) were 60 or older. The disease stages at diagnosis were mainly distributed as follows: 62.4% of patients had stage I disease, followed by 22.8% with stage II, 9.4% with stage III, and 5.3% with stage IV.

**Table 1:** Baseline data sheet for UCEC patients.

Characteristic	Levels	Overall
n		543
Clinical stage, n (%)	Stage I	339 (62.4%)
	Stage II	51 (9.4%)
	Stage III	124 (22.8%)
	Stage IV	29 (5.3%)
Primary therapy outcome, n (%)	PD	20 (4.2%)
	SD	6 (1.3%)
	PR	12 (2.5%)
	CR	436 (92%)
Age, n (%)	<=60	206 (38.1%)
	>60	334 (61.9%)
Histological type, n (%)	Endometrioid	407 (75%)
	Mixed	22 (4.1%)
	Serous	114 (21%)
Residual tumor, n (%)	R0	372 (90.7%)
	R1	22 (5.4%)
	R2	16 (3.9%)
Histologic grade, n (%)	G1	98 (18.4%)
	G2	120 (22.6%)
	G3	314 (59%)
Tumor invasion(%), n (%)	<50	259 (55.1%)
	>=50	211 (44.9%)
OS event, n (%)	Alive	452 (83.2%)
	Dead	91 (16.8%)

Regarding the primary treatment outcomes for UCEC, 92% of patients achieved a complete response, 2.5% had a partial response, 4.2% had progressive disease, and 1.3% had stable disease. Most of the analyzed tissues (75%) were endometrioid, while 21% were serous. In terms of residual tumor, 90.7% of patients had no residual tumor, and 9.3% had residual tumor. For histological grade, 18.4% of UCEC patients had high differentiation (G1), 22.6% had moderate differentiation (G2), and 59% had low differentiation (G3).

### Clinical correlation analysis of SLC2A1 and MPST

To better understand the significance and mechanisms of SLC2A1 and MPST expression in Uterine Corpus Endometrial Carcinoma (UCEC), we investigated the relationship between the expression levels of these proteins and various clinical features.

**Figure 2:**
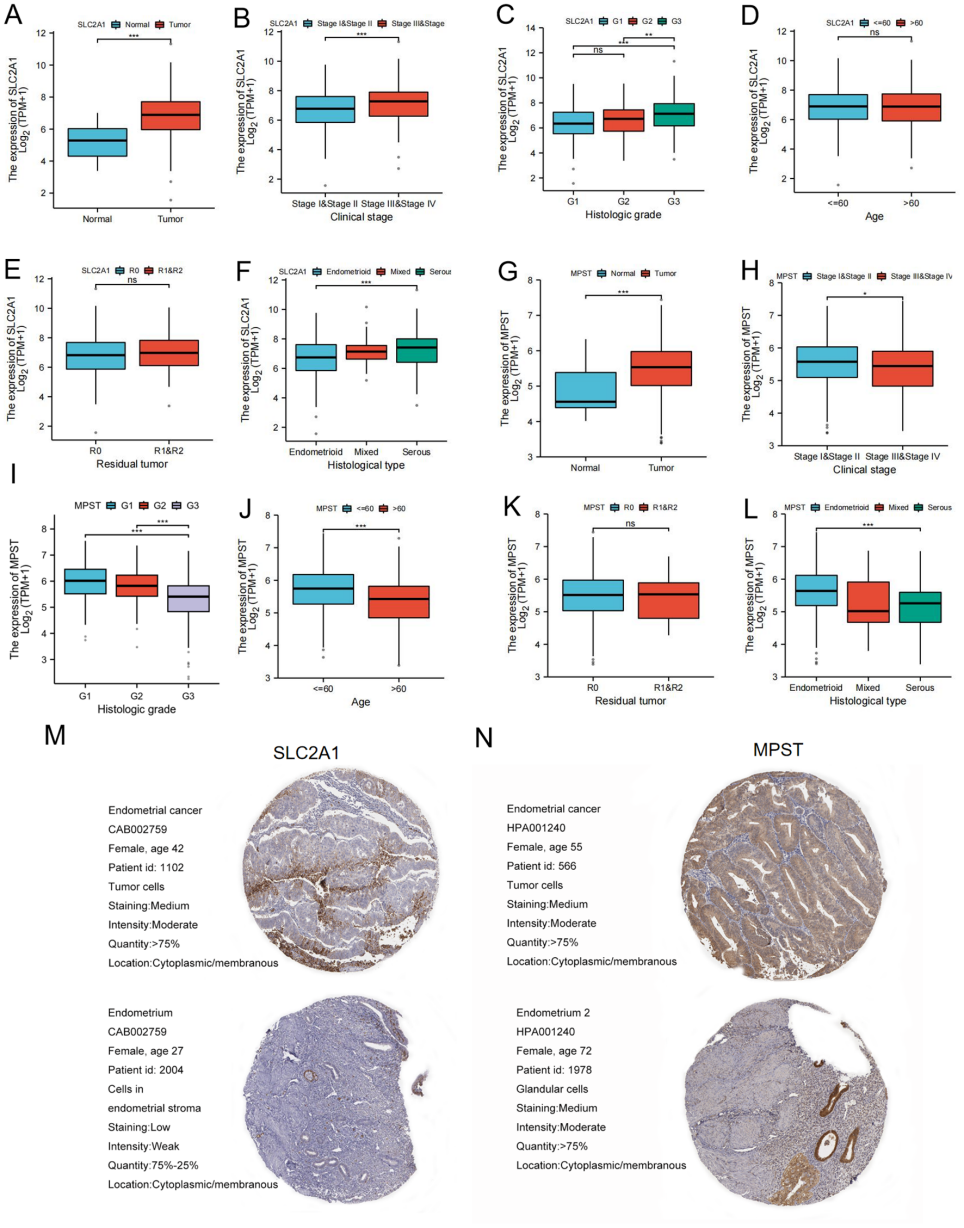
SLC2A1 and MPST expression levels correlate with multiple clinicopathological characteristics of UCEC patients. **(A–F)** The correlation analysis between SLC2A1 expression levels and **(A)** Expression level, **(B)** clinical stages, **(C)** histological grade, **(D)** age, **(E)** residual tumor, **(F)** Histological type of UCECpatients; **(G–L)** The correlation analysis between MPST expression levels and **(G)** Expression level, **(H)** clinical stages, **(I)** histological grade, **(J)** age, **(K)** residual tumor, **(L)** Histological type of UCEC patients *P < 0.05, **P <0.01,***P<0.001; **(M, N)** Immunohistochemical analysis of UCEC and normal liver tissue determined by HPA database. ns stands for not statistically significant.

Our results showed that SLC2A1 expression was up - regulated in UCEC tissues compared to normal tissues (**Figure 2A**). Correlation analysis demonstrated significant differences in SLC2A1 expression levels in relation to clinical stage (**Figure 2B**), histological type (**Figure 2C**), and histological grade (**Figure 2F**). However, there were no significant differences associated with age or tumor remnant size (**Figures 2D, E**).

Similarly, MPST expression was higher in UCEC tissues than in normal tissues (**Figure 2G**), as shown in **Figure 2**. Correlation analysis indicated that MPST expression levels differed significantly according to clinical stage (**Figure 2H**), histological type (**Figure 2L**), histological grade (**Figure 2I**), and age (**Figure 2J**), but not according to tumor remnant size (**Figure 2K**).

Immunohistochemical staining data from the HPA database further confirmed the higher expression of SLC2A1 and MPST in tumor tissues compared to adjacent normal endometrial tissues (**Figures 2M, N**).

### Differences in expression of SLC2A1 and MPST in UCEC

Following the database analysis, we used quantitative Polymerase Chain Reaction (qPCR) to measure the expression levels of SLC2A1 and MPST in tissue samples from patients with Uterine Corpus Endometrial Carcinoma (UCEC). The qPCR results revealed that both SLC2A1 and MPST were significantly up - regulated in UCEC tissues compared to normal tissues.

**Figure 3:**
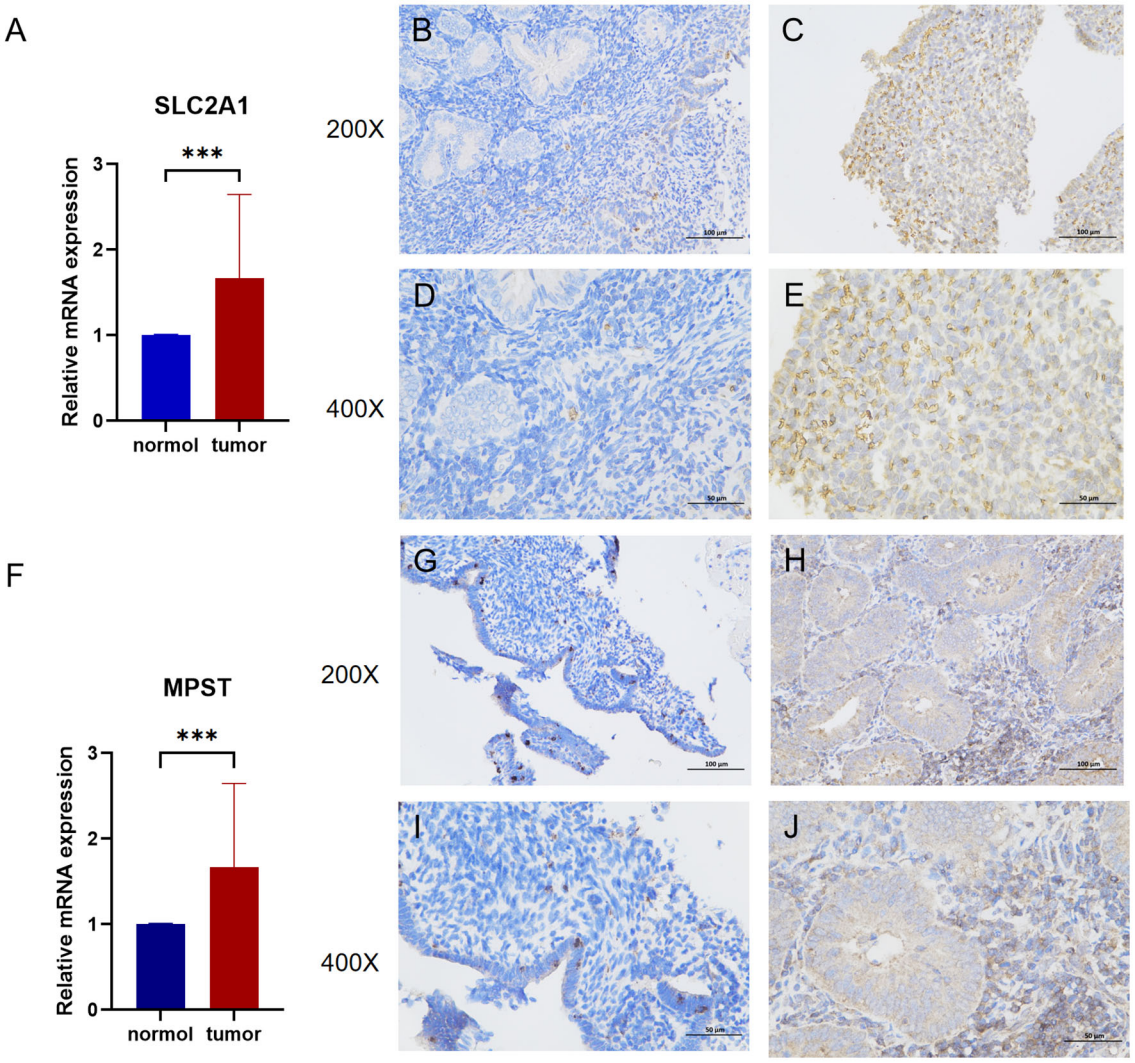
**(A)** The mRNA relative expression of SLC2A1 detected by q-PCR in UCEC and adjacent nontumor tissues (*p*=0.0009). **(B, C)** Enlarge the immunohistochemical images of SLC2A1 in normal tissue and endometrial cancer by 200 times. **(D, E)** Enlarge the immunohistochemical images of SLC2A1 in normal tissue and endometrial cancer by 400 times. **(F)** The mRNA relative expression of MPST detected by q-PCR in UCEC and adjacent nontumor tissues (*p*=0.0016). **(G, H)** Enlarge the immunohistochemical images of MPST in normal tissue and endometrial cancer by 200 times. **(I, J)** Enlarge the immunohistochemical images of MPST in normal tissue and endometrial cancer by 400 times. ***P<0.001.

Moreover, we employed immunohistochemistry to compare the protein expression levels of SLC2A1 and MPST between normal and UCEC tissues (**Figure 3**).

### Prognostic analysis

**Figure 4:**
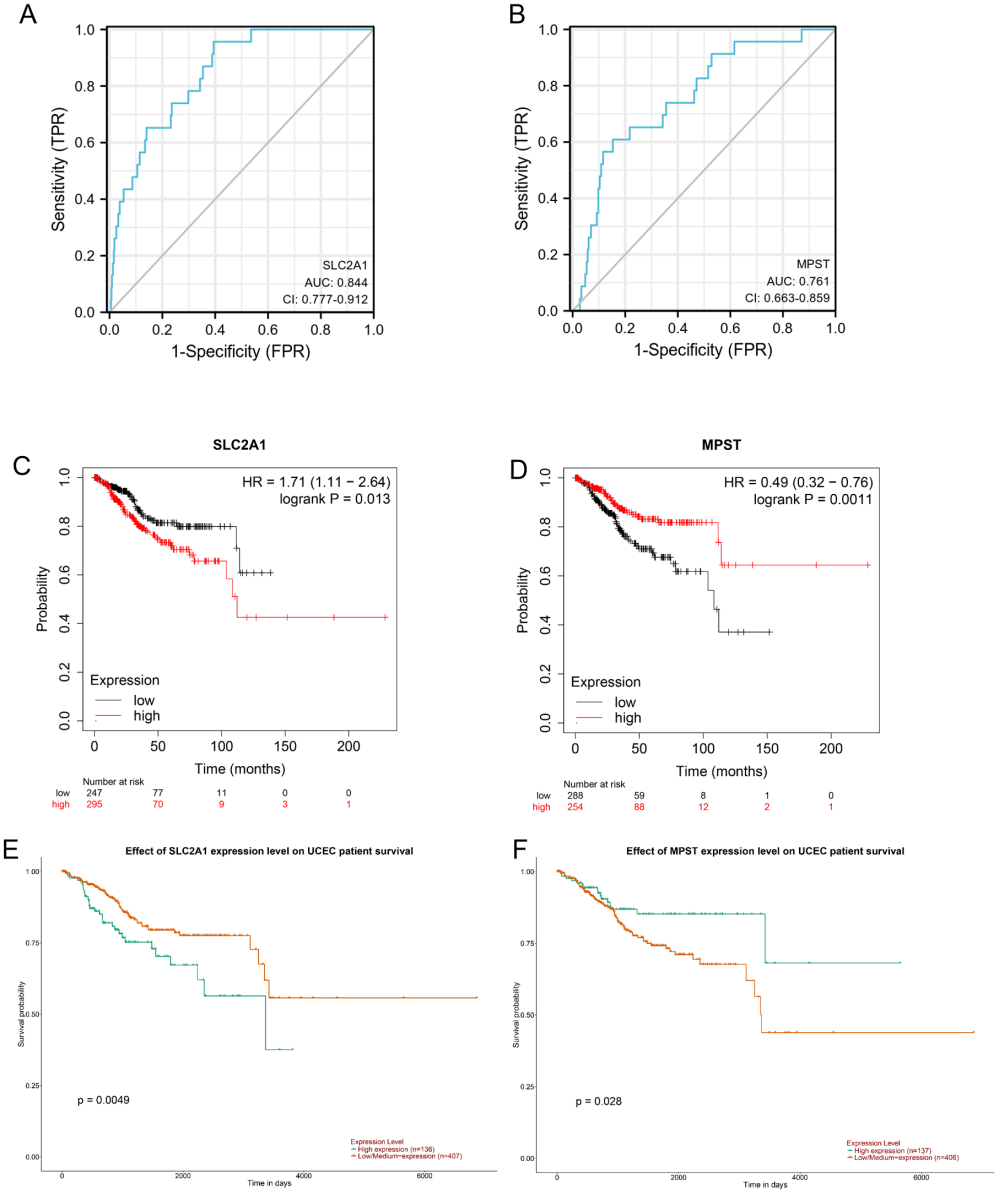
**(A, B)** Diagnostic ROC curves to distinguish UCEC tissues and normal tissues based on the SLC2A1 and MPST expression levels. Based on UALCAN database analysis, **(C)** OS survival analysis of SLC2A1, **(D)** OS survival analysis of MPST; Kaplan-Meier plotter database analysis showed that Kaplan-Meier curves showed overall survival of patients in high-and low-risk groups; **(E)** OS survival analysis of SLC2A1; **(F)** OS survival analysis of MPST.

**Figure 4** demonstrates the diagnostic potential of the SLC2A1 and MPST genes in distinguishing between benign and malignant tissues. The area under the receiver operating characteristic curve (AUC) quantifies a model’s diagnostic efficacy. An AUC of 0.5 implies a random prediction, similar to chance. As per standard evaluation criteria (1), an AUC in the range of 0.7 - 0.85 indicates fair diagnostic ability, while 0.85 - 0.95 shows good performance.​

Our study found that SLC2A1 has strong discriminatory power, with an AUC of 0.844 (**Figure 4A**), suggesting good diagnostic value. MPST also showed significant diagnostic value, with an AUC of 0.761 for its ROC curve (**Figure 4B**). These values highlight their important roles in the diagnosis of UCEC (20). Furthermore, the Kaplan - Meier survival curves indicated that high expression of MPST is associated with a better overall survival (OS) prognosis (**Figure 4F**), while low expression of SLC2A1 is linked to an improved OS prognosis (**Figure 4E**). The OS survival analysis using the UALCAN database supported these results (**Figure 4C, D**).

**Figure 5:**
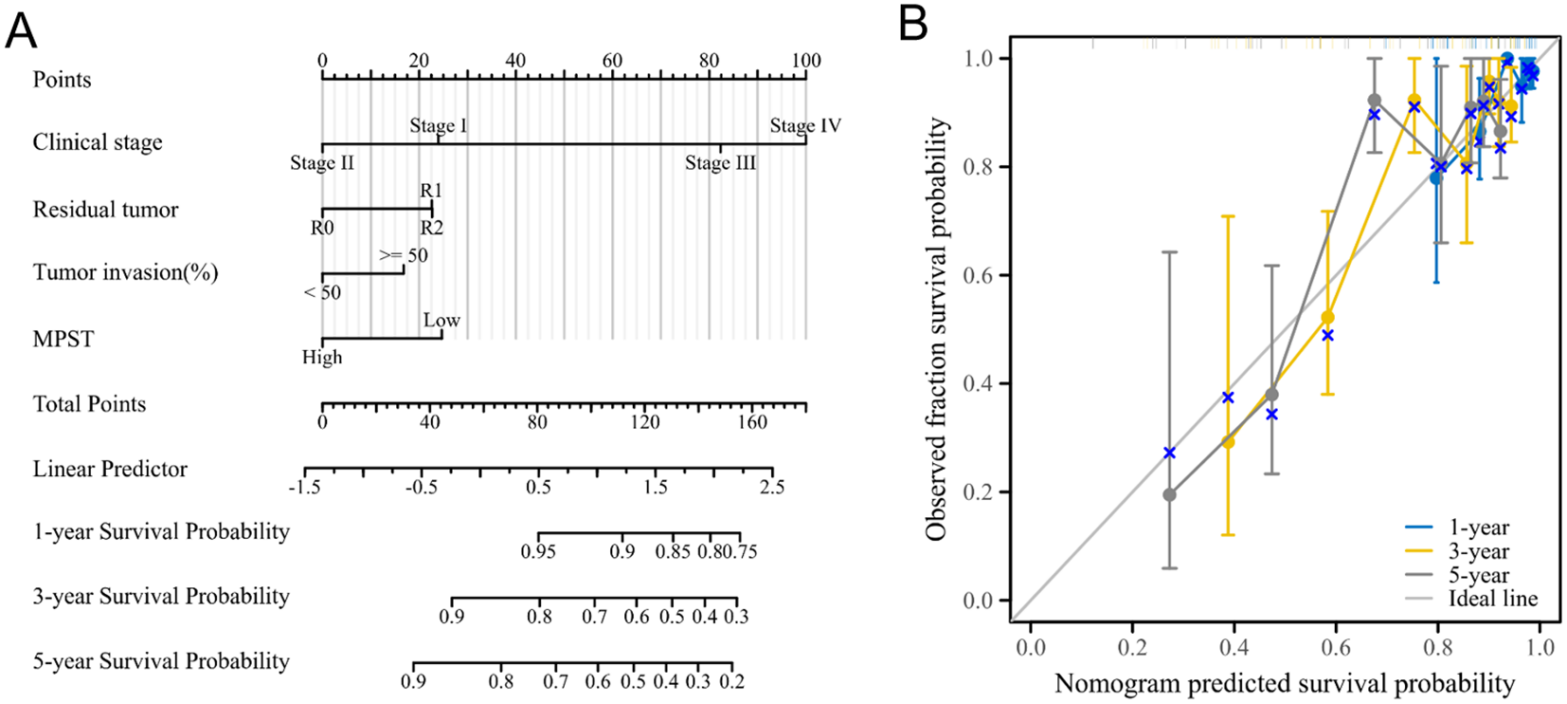
Show the superior diagnostic and prognostic performance of MPST using a nomogram model. **(A)** A nomogram model for MPST expression in UCEC; **(B)** a nomogram model for calibration curve evaluation of MPST at 1, 3 and 5 years.

Meanwhile, the nomogram model showed that MPST has superior diagnostic and prognostic performance. **Figure 5A** shows the expression pattern of MPST in UCEC, and **Figure 5B** presents a nomogram model for evaluating the calibration curves of MPST at 1, 3, and 5 years.

### Construction and evaluation of the nomogram model

To explore the impact of MPST expression on endometrial cancer prognosis in depth, we performed a univariate Cox regression analysis centered on MPST (**Table 2**). Using the analysis results, we carefully constructed a nomogram model to verify its accuracy and practicality in prognosis assessment. Additionally, calibration curves were used to comprehensively evaluate the precision of the nomogram model in predicting 1 - year, 3 - year, and 5 - year survival rates. The findings clearly show the significant predictive potential of MPST. The high accuracy of the nomogram model’s calibration curves for 1 - year, 3 - year, and 5 - year survival predictions (**Figure 5**) validates this potential.

**Table 2:** Univariate and multivariate Cox regression analysis between UCEC clinical characteristics and OS.

Characteristics	Total (N)	Univariate analysis		Multivariate analysis	
		Hazard ratio (95% CI)	*P* value	Hazard ratio (95% CI)	*P* value
Clinical stage	553				
Stage I&Stage II	394	Reference		Reference	
Stage III&Stage IV	159	3.553 (2.362 - 5.344)	**< 0.001**	4.392 (2.443 - 7.896)	**< 0.001**
Residual tumor	414				
R0	376	Reference		Reference	
R1&R2	38	3.112 (1.774 - 5.459)	**< 0.001**	1.951 (1.022 - 3.725)	**0.043**
Tumor invasion(%)	475				
< 50	261	Reference		Reference	
>= 50	214	2.825 (1.752 - 4.554)	**< 0.001**	1.381 (0.771 - 2.473)	0.278
MPST	553				
Low	277	Reference		Reference	
High	276	0.640 (0.425 - 0.964)	**0.033**	0.567 (0.335 - 0.961)	**0.035**

The bold numbers indicate statistical significance.

### PPI network and enrichment analysis in cancer

**Figure 6:**
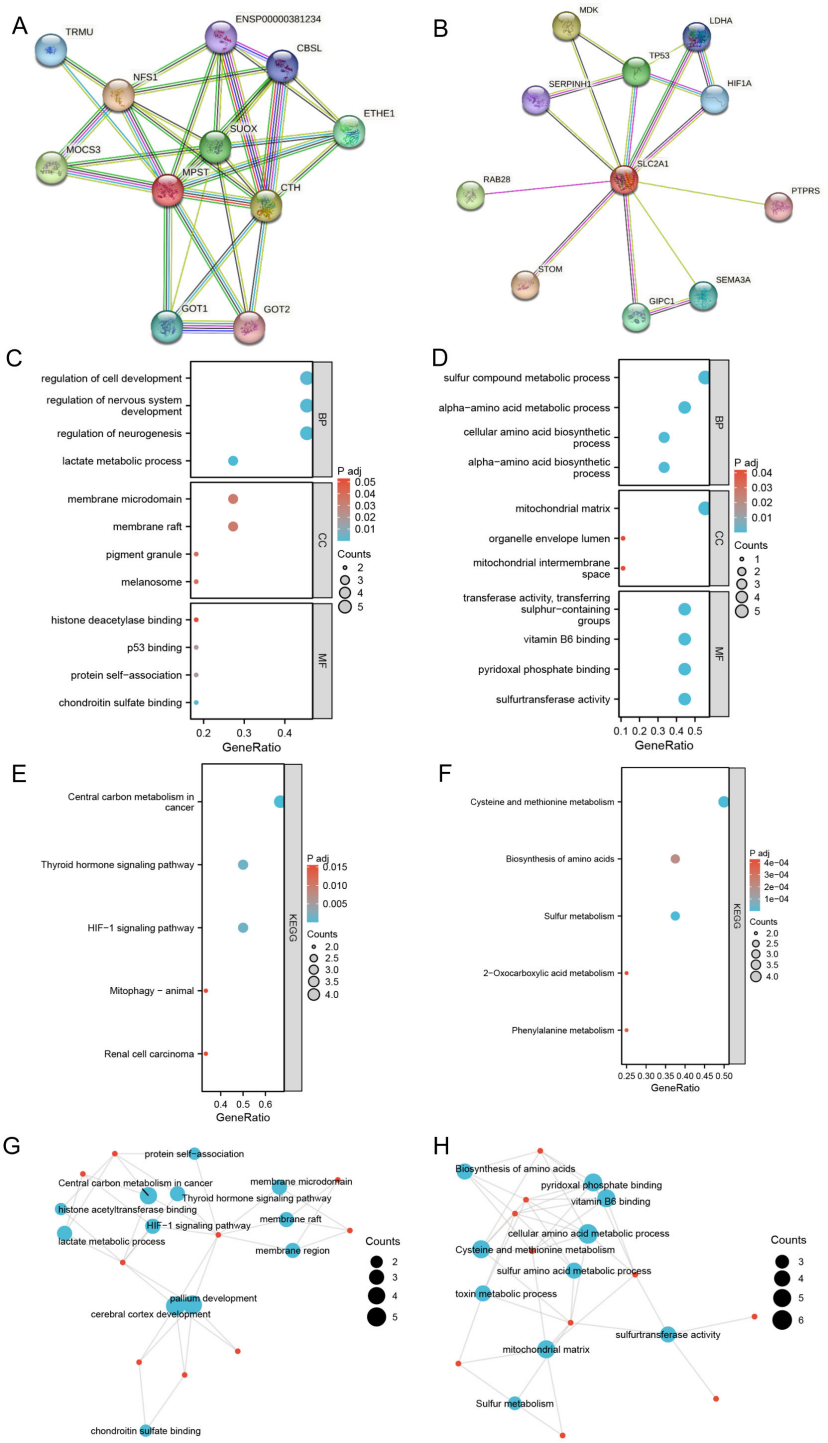
Construct PPI network and make enrichment analysis. **(A, B)** Synthesis analysis of protein-protein interaction of SLC2A1 and MPST; **(C, D)** GO enrichment show the enriched biological functions (BP), cellular components (CC), and molecular functions (MF); **(E, F)** KEGG pathway enrichment analysis of SLC2A1 and MPST; **(G, H)** GO term and KEGG pathway enrichment analysis of SLC2A1and MPST.

We analyzed components such as membrane microdomains, membrane rafts, and pigment granules (**Figures 6A, B**). Among the identified molecular functions, chondroitin sulfate binding, protein self-association, p53 binding, and histone deacetylase binding were the most common. KEGG pathway enrichment analysis showed significant correlations with multiple pathways, including renal cell carcinoma, mitophagy in animals, the HIF-1 signaling pathway, the thyroid hormone signaling pathway, and central carbon metabolism in cancer (see **Figures 6C, E, G**).

Regarding MPST, the key biological processes observed were the alpha-amino acid biosynthetic process, cellular amino acid biosynthetic process, alpha-amino acid metabolic process, and sulfur compound metabolic process. The most enriched cellular components were the mitochondrial intermembrane space, organelle capsule cavity, and mitochondrial matrix. The predominant molecular functions included sulfurtransferase activity, pyridoxal phosphate binding, vitamin B6 binding, and transfer activity related to the transfer of sulfur-containing groups. KEGG pathway enrichment analysis uncovered significant associations with various pathways, such as phenylalanine metabolism, 2-oxalic acid metabolism, sulfur metabolism, amino acid biosynthesis, and cysteine and methionine metabolism (see **Figures 6D, F, H**). The GO and KEGG enrichment analyses of the MPST and SLC2A1 genes in UCEC, together with their functional partner genes, are elaborated in **Tables 3** and **4**.

**Table 3:** GO and KEGG enrichment analyses of SLC2A1 and functional partner genes in UCEC.

Ontology	ID	Description	*P* value
BP	GO:0006089	lactate metabolic process	6.05e-08
BP	GO:0050767	regulation of neurogenesis	5.91e-07
BP	GO:0051960	regulation of nervous system development	1.57e-06
BP	GO:0060284	regulation of cell development	2.94e-06
BP	GO:0045926	negative regulation of growth	5.56e-06
CC	GO:0045121	membrane raft	0.0005
CC	GO:0098857	membrane microdomain	0.0005
CC	GO:0042470	melanosome	0.0013
CC	GO:0048770	pigment granule	0.0013
CC	GO:0072562	blood microparticle	0.0024
MF	GO:0035374	chondroitin sulfate binding	1.19e-05
MF	GO:0043621	protein self-association	0.0005
MF	GO:0002039	p53 binding	0.0006
MF	GO:0042826	histone deacetylase binding	0.0020
MF	GO:0008201	heparin binding	0.0036
KEGG	hsa05230	Central carbon metabolism in cancer	2.57e-08
KEGG	hsa04066	HIF-1 signaling pathway	2.35e-05
KEGG	hsa04919	Thyroid hormone signaling pathway	3.21e-05
KEGG	hsa04137	Mitophagy - animal	0.0007
KEGG	hsa05211	Renal cell carcinoma	0.0007

**Table 4:** GO and KEGG enrichment analyses of MPST and functional partner genes in UCEC.

Ontology	ID	Description	*P* value
BP	GO:0006790	sulfur compound metabolic process	6.87e-06
BP	GO:0043650	dicarboxylic acid biosynthetic process	1.44e-05
BP	GO:0002098	tRNA wobble uridine modification	1.66e-05
BP	GO:0006103	2-oxoglutarate metabolic process	2.15e-05
BP	GO:0002097	tRNA wobble base modification	2.42e-05
CC	GO:0005759	mitochondrial matrix	4.23e-07
CC	GO:0005758	mitochondrial intermembrane space	0.0334
CC	GO:0031970	organelle envelope lumen	0.0374
MF	GO:0016783	sulfurtransferase activity	3.07e-12
MF	GO:0016782	transferase activity, transferring sulphur-containing groups	1.25e-08
MF	GO:0030170	pyridoxal phosphate binding	1.4e-06
MF	GO:0070279	vitamin B6 binding	1.48e-06
MF	GO:0019842	vitamin binding	2.77e-05
KEGG	hsa00270	Cysteine and methionine metabolism	9.16E-10
KEGG	hsa00920	Sulfur metabolism	1.14E-07
KEGG	hsa01230	Biosynthesis of amino acids	8.35E-07
KEGG	hsa00360	Phenylalanine metabolism	0.0001
KEGG	hsa01210	2-Oxocarboxylic acid metabolism	0.0002

### The connection between SLC2A1, MPST, and immune cells

**Figure 7:**
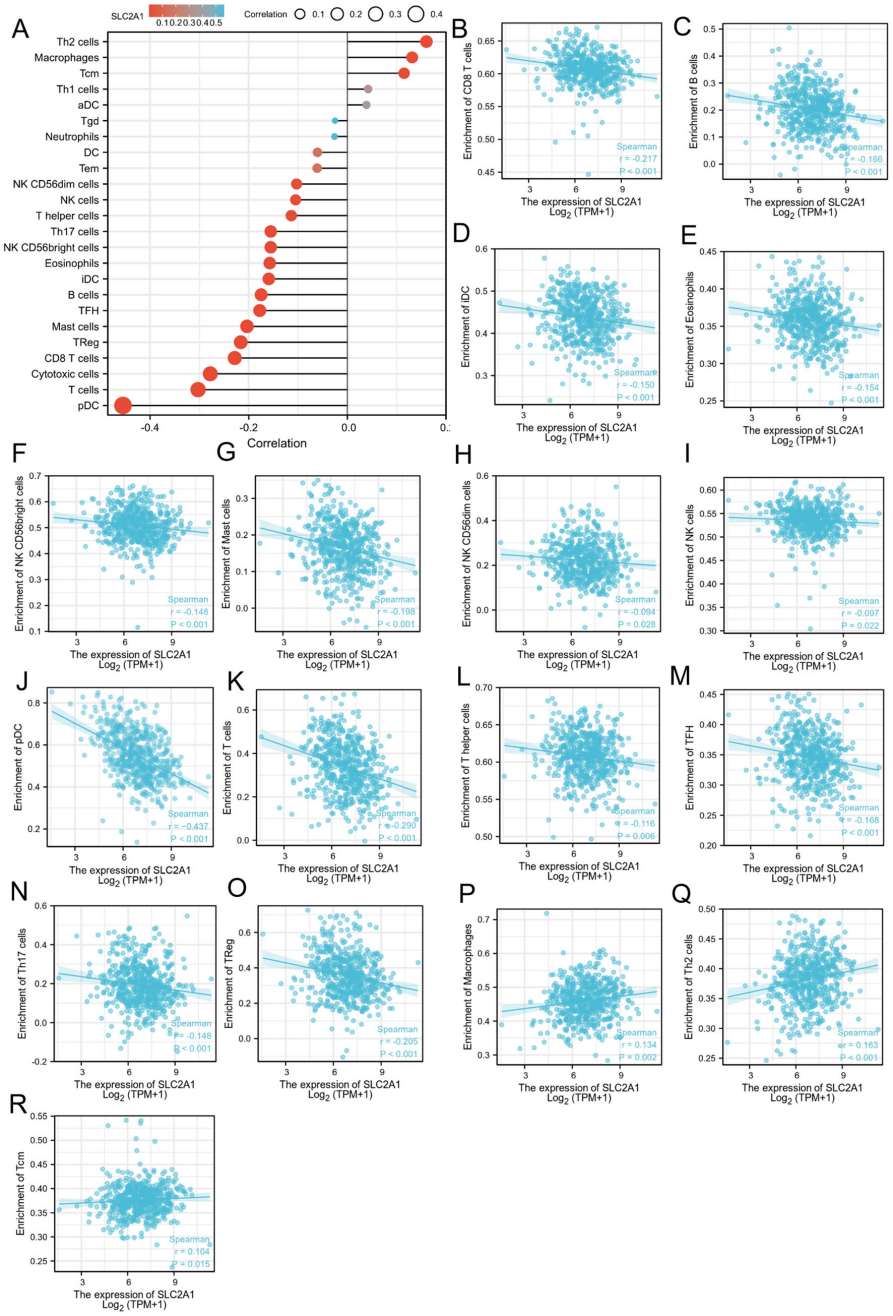
The relation of SLC2A1 and immune cells. **(A)**The relationship between SLC2A1 expression and immune cell infiltration was analyzed based on TCGA database; **(B–R)**. The correlation analysis results between the expression levels of SLC2A1 and the expression levels of some immune cells in the TCGA-UCEC dataset.

We found that the expression level of SLC2A1 was inversely correlated with the levels of various immune cell types (**Figure 7A**). These included CD8 T cells (**Figure 7B**), B cells (**Figure 7C**), immature dendritic cells (iDC) (**Figure 7D**), eosinophils (**Figure 7E**), CD56bright natural killer (NK) cells (**Figure 7F**), mast cells (**Figure 7G**), CD56dim NK cells (**Figure 7H**), NK cells (**Figure 7I**), plasmacytoid dendritic cells (pDC) (**Figure 7J**), T cells (**Figure 7K**), T helper cells (**Figure 7L**), T follicular helper (TFH) cells (**Figure 7M**), Th17 cells (**Figure 7N**), and regulatory T cells (TReg) (**Figure 7O**). In contrast, the expression of SLC2A1 was positively correlated with the levels of macrophages (**Figure 7P**), Th2 cells (**Figure 7Q**), and central memory T cells (Tcm) (**Figure 7R**).

**Figure 8:**
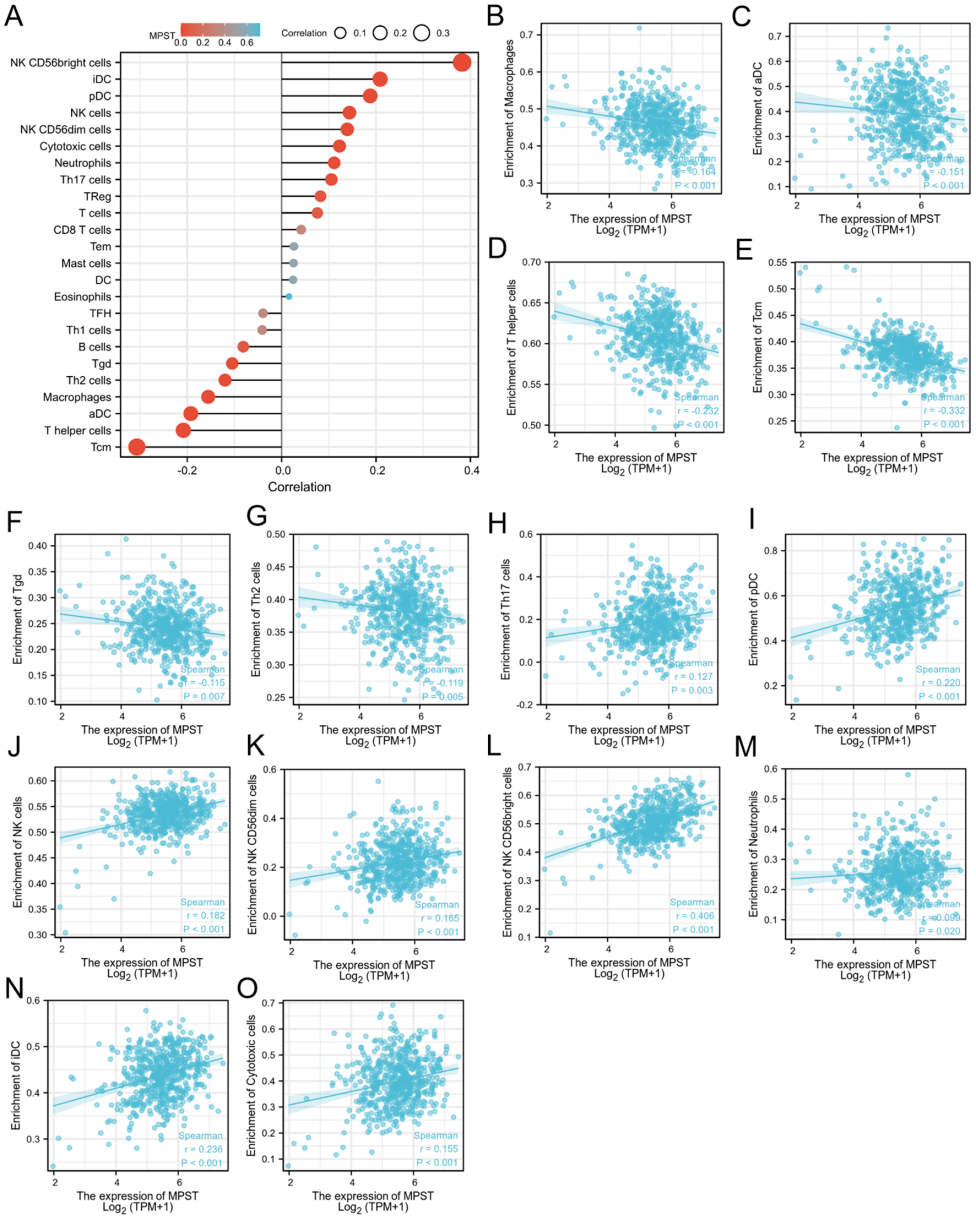
The relation of MPST and immune cells. **(A)** The relationship between MPST expression and immune cell infiltration was analyzed based on TCGA database; **(B-O)** The correlation analysis results between the expression levels of MPST and the expression levels of some immune cells in the TCGA-UCEC.

As for MPST, its expression levels showed an inverse correlation with those of macrophages (**Figure 8B**), activated dendritic cells (aDC) (**Figure 8C**), T helper cells (**Figure 8D**), Tcm (**Figure 8E**), γδ T cells (Tgd) (**Figure 8F**), and Th2 cells (**Figure 8G**). Moreover, the expression of MPST was positively correlated with Th17 cells (**Figure 8H**), pDC (**Figure 8I**), NK cells (**Figure 8J**), CD56dim NK cells (**Figure 8K**), CD56bright NK cells (**Figure 8L**), neutrophils (**Figure 8M**), iDC (**Figure 8N**), and cytotoxic cells (**Figure 8O**). **Figure 8A** illustrates the correlation between MPST expression and immune cell infiltration.

### Important immune genes associated with tumor immune escape

**Figure 9:**
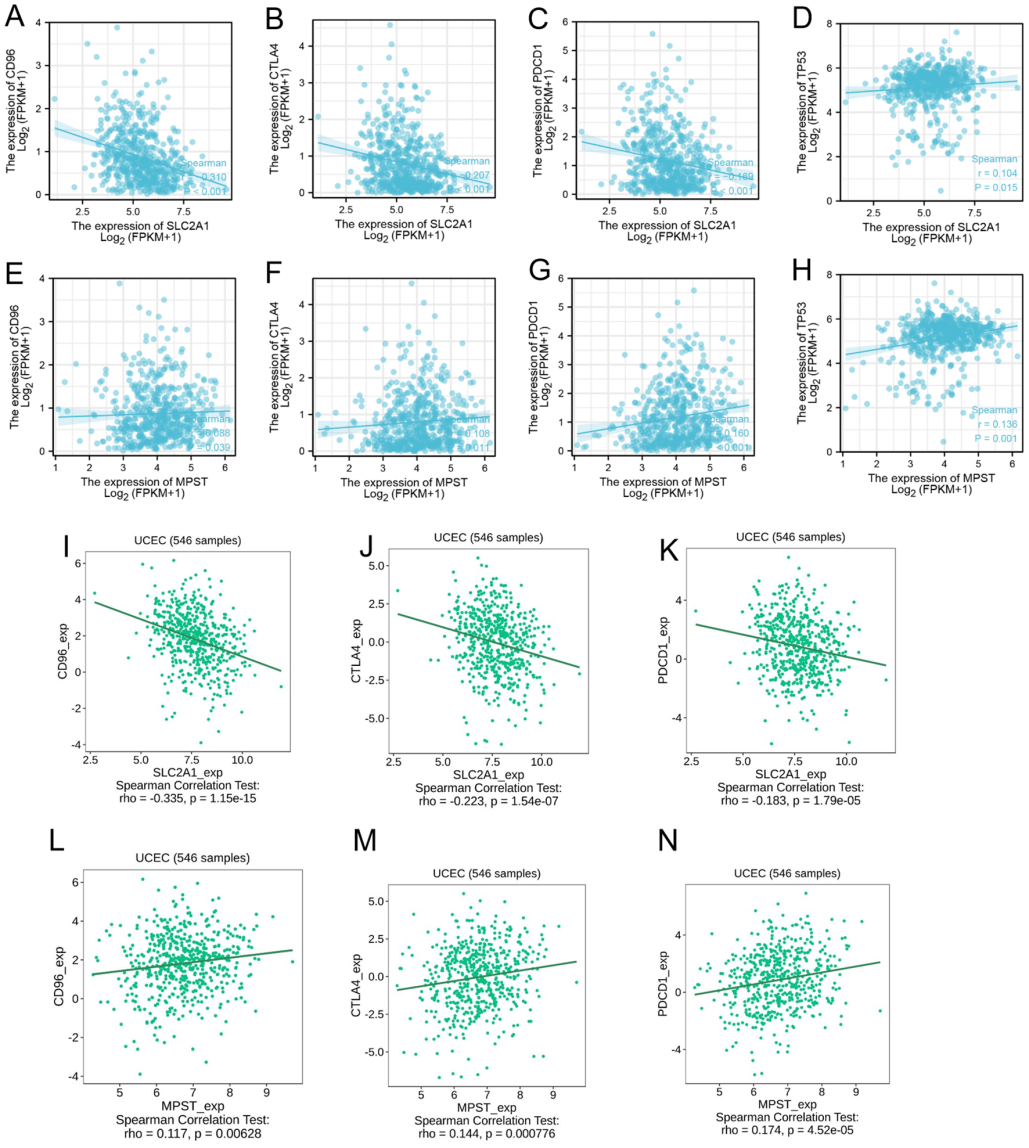
The expression level of SLC2A1 **(A–D)** and MPST **(E–H)** is associated with CD96, CTLA-4, PDCD-1 and TP53 in tumor immune escape; **(I–N)** The same results in the TISIDIB database.

CD96, CTLA-4, and PDCD-1 are important immune checkpoint proteins that play a crucial role in tumor immune evasion. Through our analysis of the TCGA dataset, we discovered that in Uterine Corpus Endometrial Carcinoma (UCEC) samples, the expression level of SLC2A1 was negatively correlated with the expression levels of CD96, CTLA-4, and PDCD-1 (**Figures 9A–C**). On the contrary, the expression level of MPST showed a positive correlation with those of CD96, CTLA-4, and PDCD-1 (**Figures 9E–G**).

The TP53 tumor suppressor gene is usually expressed at low levels in normal cells, while its expression is significantly elevated in malignant tumors. Our observations indicated that both SLC2A1 and MPST were positively correlated with the expression level of TP53 (**Figures 9D, H**). Moreover, these results were further validated by using the TISIDB database (**Figures 9I–N**).

### Correlation analysis of the methylation levels of SLC2A1 and MPST with UCEC

**Figure 10:**
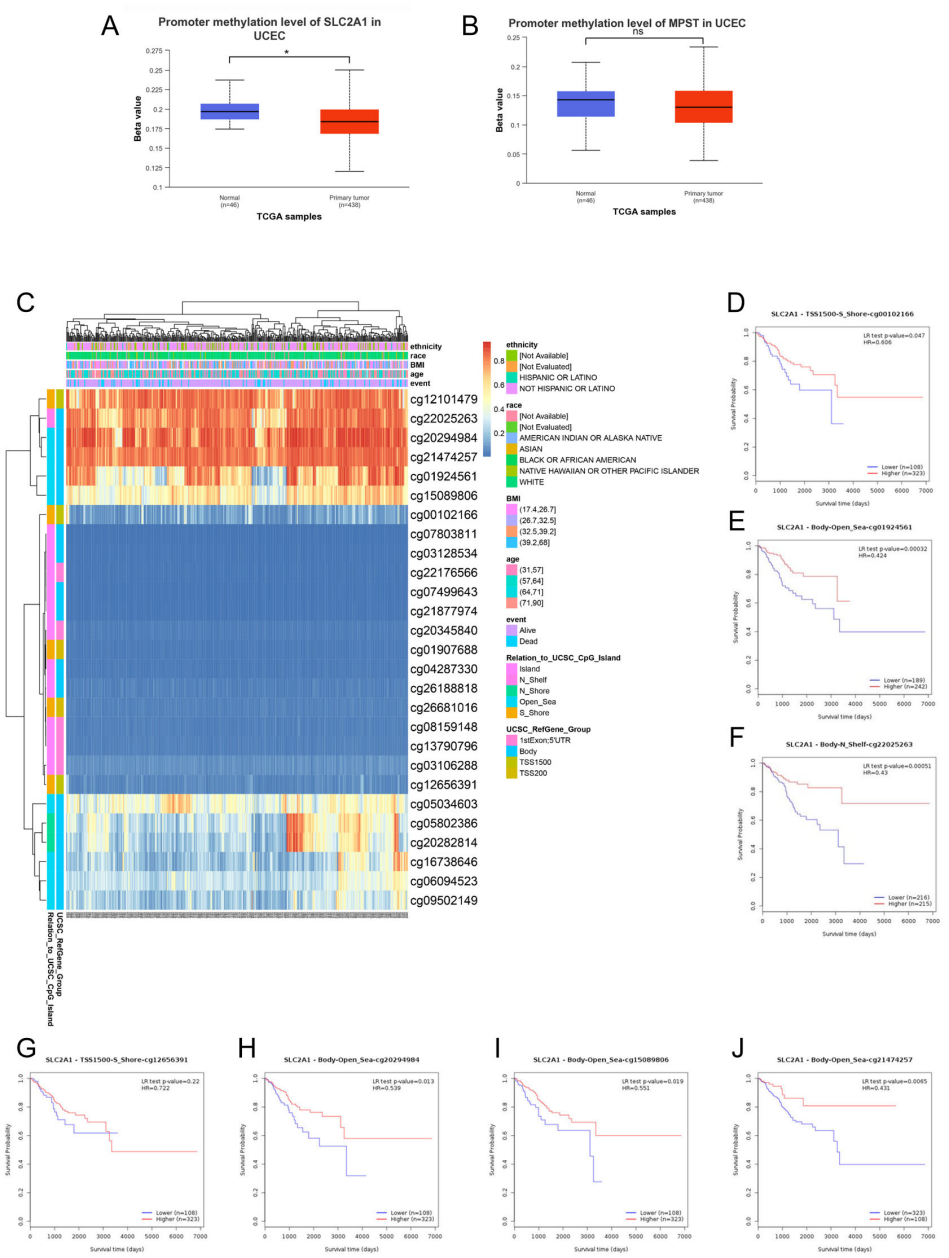
DNA methylation levels in the SLC2A1 gene are associated with the prognosis of UCEC patients. DNA methylation levels in the SLC2A1 and MPST gene **(A, B)**.The heat map showed the SLC2A1 DNA methylation at CpG sites **(C)**. **(D–J)** the Kaplan–Meier (K–M) curves of overall survival (OS) shows the difference between the low and high expression of SLC2A1 methylation of cg00102166 **(D)**, cg01924561 **(E)**, cg22025263 **(F)**, cg12656391 **(G)**, cg20294984 **(H)**, cg15089806 **(I)**, cg21474257 **(J)** CpG sites in UCEC.

Promoter DNA methylation has been demonstrated to influence transcriptional repression and contribute to tumorigenesis. Given that functional enrichment analysis suggested that SLC2A1 was likely involved in the methylation process, we then analyzed the methylation status of SLC2A1 in relation to MPST expression. We compared the methylation values of SLC2A1 and MPST between normal and tumor tissues. As depicted in **Figure 10A**, the methylation values of SLC2A1 were significantly reduced (*P* < 0.05), whereas there was no significant difference for MPST (*P* > 0.05) (**Figure 10B**). This finding implies that the transcriptional expression of SLC2A1 might be associated with promoter hypomethylation.

We further utilized the MetSurv tool to analyze the DNA methylation level within the SLC2A1 gene and the prognostic significance of CpG islands in the SLC2A1 gene. The results showed that there were 27 methylated CpG islands in SLC2A1, such as cg07803811, cg03128534, cg22176566, cg07499643, cg21877974, cg20345840, cg01907688, cg04287330, cg26188818, cg26681016, cg08159148, cg13790796, cg03106288, and cg12656391, all of which exhibited decreased DNA methylation levels (**Figure 10C**).

Moreover, the MethSurv analysis indicated that patients with lower overall survival had lower SLC2A1 methylation levels compared to those with higher SLC2A1 methylation (P < 0.05) (**Table 5**). We identified seven CpG sites located on the CpG islands that were associated with a poor prognosis, namely cg00102166, cg01924561, cg12656391, cg15089806, cg20294984, cg21474257, and cg22025263 (**Figures 10D–J**). In comparison to patients with higher CpG methylation in SLC2A1, the reduced SLC2A1 methylation at these seven CpG islands was associated with poorer overall survival in UCEC patients.

**Table 5:** Effects of methylation levels in the CpG sites of the SLC2A1 gene on the prognosis of UCEC patients.

Name	HR	*P* value
Cg00102166	0.606	0.04
Cg01907688	1.734	0.082
Cg01924561	0.424	0.0004
Cg03106288	1.455	0.21
Cg03128534	1.697	0.024
Cg04287330	1.826	0.049
Cg05034603	1.389	0.19
Cg05802386	0.625	0.13
Cg06094523	1.181	0.56
Cg07499643	1.58	0.053
Cg07803811	0.823	0.5
Cg08159148	1.968	0.0047
Cg09502149	0.756	0.27
Cg12101479	0.645	0.13
Cg12656391	0.491	0.025
Cg13790796	1.658	0.034
Cg15089806	0.551	0.015
Cg16738646	1.103	0.68
Cg20282814	0.615	0.1
Cg20294984	0.539	0.01
Cg20345840	2.745	0.0046
Cg21474257	0.431	0.014
Cg21877974	1.684	0.067
Cg22025263	0.43	0.0008
Cg22176566	1.653	0.09
Cg26188818	0.638	0.16
Cg26681016	1.539	0.16

### Genetic alterations in SLC2A1 and mpst are not connected with survival outcomes in UCEC patients

We conducted an investigation of the SLC2A1 and MPST genes using 549 Uterine Corpus Endometrial Carcinoma (UCEC) samples with mutations. Genetic alterations in the SLC2A1 gene were detected in only 4% of UCEC patients (**Supplementary Figure S1A**). According to the Kaplan-Meier survival curve analysis, there was no significant difference in overall survival (OS) among these patients (*P* = 0.809) (**Supplementary Figure S1C**). Similarly, alterations in the MPST gene were found in merely 1.7% of UCEC patients (**Supplementary Figure S1B**), and the Kaplan-Meier survival curve analysis also indicated no significant difference in OS (*P* = 0.587) (**Supplementary Figure S1D**). In conclusion, there was no notable disparity in overall survival between patients with and without genetic changes in either the SLC2A1 or MPST genes.

## Discussion

In our study, we detected a significant overexpression of SLC2A1 in 22 out of 33 human cancer tissues, while MPST was upregulated in 13 out of 33 tumor tissues. Both SLC2A1 and MPST showed high expression in endometrial cancer clinical samples and in the TCGA database. The SLC2A1 gene encodes a glucose transporter of the solute carrier family (GLUT), which initiates glucose utilization. Previous research has identified SLC2A1 as a potential prognostic biomarker for immunotherapy in lung adenocarcinoma (21). In colorectal cancer, METTL3 has been shown to have an oncogenic role by stabilizing HK2 and SLC1A2 mRNA via the IGF2BPs axis, regulating glycolytic metabolism and cell proliferation (22). Based on these findings, SLC2A1 and MPST may serve as diagnostic markers for uterine corpus endometrial carcinoma (UCEC).

Our research revealed significant associations between SLC2A1 expression in UCEC tissues and clinical stage, histological type, histological grade, and overall survival. Similarly, MPST expression was remarkably correlated with age, histological type, histological grade, clinical stage, and overall survival. Higher SLC2A1 expression was linked to a poorer prognosis in UCEC patients, while lower MPST expression was associated with a poorer prognosis. Kaplan - Meier survival curves demonstrated that higher SLC2A1 expression correlated with lower overall survival, and lower MPST expression correlated with higher overall survival. The diagnostic values for UCEC were 0.844 and 0.761 for SLC2A1 and MPST, respectively. We validated these results using quantitative PCR (q - PCR) and immunohistochemistry, and the findings were consistent.

Analysis of differentially expressed genes (DEGs) related to SLC2A1 showed significant correlations with multiple cancer - related pathways, such as carbon metabolism, thyroid hormone signaling, HIF - 1 signaling, membrane rafts, cerebral cortex development, and lactate metabolism. In contrast, KEGG analysis indicated that MPST is mainly involved in cysteine and methionine metabolism, leading to homocysteine production. Homocysteine is crucial for physiological processes like cell cycle progression and cellular homeostasis maintenance (23). Thus, SLC2A1 may influence UCEC progression by modulating cancer cell metabolism, and there may be a connection between MPST and UCEC development due to its role in cysteine and methionine metabolism.

SLC2A1 (encoding glucose transporter GLTT1) plays a pivotal role in cancer metabolism through mediating the Warburg effect, a metabolic hallmark characterized by enhanced glucose uptake and preferential glycolytic flux in neoplastic cells. This oncogenic reprogramming facilitates tumor progression via multiple mechanisms (1): Accelerated glycolysis provides rapid ATP generation and metabolic intermediates for macromolecule biosynthesis (2); Microenvironmental adaptation through hypoxia-inducible factor 1α (HIF-1α)-mediated regulation (3); Promotion of metastatic potential via PI3K/AKT pathway activation. Our pathway analysis in endometrial cancer (EC) reveals SLC2A1’s involvement in diverse metabolic networks, suggesting its crucial role in tumor-specific metabolic reprogramming. Notably, MPST (mercaptopyruvate sulfurtransferase) demonstrates parallel significance in oncogenic metabolism. While previous studies established its tumor-promoting effects through hydrogen sulfide (H2S)-mediated apoptosis inhibition and angiogenesis promotion, our current investigation identifies its novel association with cysteine/methionine metabolic pathways in EC. This finding positions MPST as a potential key regulator of sulfur amino acid metabolism in endometrial carcinogenesis. Future research directions should focus on experimental validation using uterine corpus endometrial carcinoma (UCEC) models. Mechanistic studies employing CRISPR/Cas9-mediated gene silencing and ectopic expression approaches are warranted to (1): Elucidate the crosstalk between SLC2A1-mediated glucose metabolism and MPST-regulated sulfur pathways (2); Characterize their combinatorial effects on malignant proliferation, invasion capacity, and chemoresistance (3); Investigate microenvironmental remodeling through systematic analysis of immune cell infiltration patterns and cytokine profiles. These investigations will provide critical insights into metabolic vulnerabilities for therapeutic targeting in UCEC.

Our research provided compelling evidence of a potential link between SLC2A1 and MPST expression and immune cell infiltration. SLC2A1 expression was negatively correlated with various immune cell types, including B cells, T cells, CD56dim cells, eosinophils, immature dendritic (iDC) cells, mast cells, natural killer (NK) cells, CD56bright cells, and plasmacytoid dendritic (pDC) cells. Similarly, MPST expression was inversely related to macrophages, T helper cells, central memory T cells (Tcm), Th2 cells, and T gamma delta (Tgd) cells. M1 macrophages possess antitumor properties, and NK cells can induce tumor cell apoptosis by interacting with Fas ligand (FasL) or tumor necrosis factor - related apoptosis - inducing ligand (TRAIL) receptors. Highly activated multifunctional CD4(+) T cells also enhance and maintain the host’s overall antitumor immunity (24). Our results suggest that overexpression of SLC2A1 and MPST helps UCEC cells evade the immune system, promoting their growth and progression.

CD96, CTLA - 4, and PDCD1 are key proteins in tumor immune evasion (25). Although CD96 inhibitors have not been tested in clinical trials yet, pre - clinical evidence shows their effectiveness in preventing cancer metastasis in various mouse models (26). TP53 is highly expressed in malignant tumors, and its mutations are associated with poor prognoses in many human cancers (27), impairing antitumor immunity and reducing the efficacy of cancer immunotherapy (28–31). We investigated the correlation between SLC2A1 and MPST expression levels and immune checkpoint genes CD96, CTLA - 4, PDCD1, and TP53. A significant correlation was found, suggesting that targeting SLC2A1 and MPST may enhance immunotherapy efficacy in UCEC patients.

DNA methylation is a key epigenetic process. It involves adding a methyl group to cytosine in DNA, mainly regulated by DNA methyltransferases (DNMTs) like DNMT1, DNMT3A, and DNMT3B (32). In normal cells, proper DNA methylation controls gene expression. Methylation in promoter regions can repress gene transcription, either by blocking transcription factor binding or recruiting proteins that condense chromatin structure (33, 34).In cancer, abnormal DNA methylation is common. Hypermethylation of promoter regions often silences tumor - suppressor genes, while hypomethylation can activate oncogenes (32–36). In our study, we found that the methylation level of the SLC2A1 gene is significantly associated with the prognosis of UCEC patients (37). Specifically, hypermethylation at specific CpG sites, namely cg00102166, cg01924561, cg12656391, cg15089806, cg20294984, cg21474257, and cg22025263, was correlated with poorer overall survival. This finding aligns with the general understanding that abnormal DNA methylation in cancer can disrupt normal gene function and contribute to disease progression.​

Previous research has also demonstrated the importance of DNA methylation in regulating the expression of genes related to cancer metabolism. For example, promoter CpG island hypermethylation - linked inactivation of DERL3, a gene involved in the endoplasmic reticulum - associated protein degradation pathway, leads to overexpression of SLC2A1 (glucose transporter 1, GLUT1). SLC2A1 overexpression, in turn, contributes to the Warburg effect, a characteristic metabolic reprogramming in cancer cells. This further emphasizes the significance of DNA methylation in modulating gene expression and cancer - related phenotypes. In this study, we observed a low incidence of mutations in SLC2A1 and MPST genes in UCEC tissues, with rates of only 4% and 1.7% respectively, and no association between these mutations and overall survival (OS) in UCEC patients. However, the methylation - related changes in SLC2A1 suggest that epigenetic mechanisms may play a more prominent role in the regulation of this gene in UCEC.

In summary, this study investigated the expression of SLC2A1 and MPST across different cancer types. Through comprehensive bioinformatics analysis of the TCGA database, we visualized the diagnostic and prognostic significance of SLC2A1 and MPST in UCEC. We also performed an integrated analysis of SLC2A1 and MPST methylation levels, exploring the impact of gene alterations on patient disease outcomes. Additionally, we experimentally validated the differential expression of SLC2A1 and MPST in UCEC tissues compared to normal tissues, confirming their potential as diagnostic markers for UCEC patients and indicating their promise as therapeutic targets for novel immunotherapy strategies.

While the current study leveraged robust bioinformatics pipelines with parameters aligned with field standards, we recognize that comparative evaluation of alternative computational models and systematic hyperparameter optimization could further enhance predictive robustness. Future investigations will employ nested cross-validation frameworks to rigorously assess model performance across parameter spaces while mitigating overfitting risks. We are currently integrating multi-omics validation cohorts (including proteomic profiles and single-cell resolution datasets) to enable architecture benchmarking across diverse machine learning paradigms. Importantly, these computational refinements will be functionally validated through parallel experimental approaches—particularly patient-derived organoid drug response assays and spatial transcriptomic mapping of tumor microenvironment dynamics. Such iterative integration of computational and experimental methodologies will strengthen the translational applicability of molecular subtyping strategies in clinical decision-making.

## Conclusion

In this study, we have firmly established the diagnostic and prognostic significance of SLC2A1 and MPST in Uterine Corpus Endometrial Carcinoma (UCEC). Specifically, SLC2A1 is overexpressed in the tumor tissues of UCEC patients, and MPST also exhibits a high level of expression in these tissues. Our findings indicate that the expression levels of SLC2A1 and MPST are correlated with the presence of immune cells within tumors. This correlation suggests that these two genes may play a pivotal role in immune response-based therapies for UCEC patients.

Mechanistically, SLC2A1 is thought to be a key player in cancer cell proliferation and metastasis through multiple pathways. These include central carbon metabolism in cancer, the HIF-1 signaling pathway, and lactic acid metabolic processes. In contrast, MPST is involved in cysteine and methionine metabolism, which contributes to the development of UCEC.

Furthermore, the expression levels of SLC2A1 and MPST in UCEC tumors are closely related to the infiltration patterns of various immune cell types. This relationship has the potential to impact the effectiveness of immune therapies. Additionally, we have found that the methylation status and gene expression of SLC2A1 are associated with the prognosis of UCEC patients.

In light of these findings, targeting SLC2A1 and MPST emerges as a promising therapeutic strategy for UCEC. Moreover, their expression levels can serve as valuable diagnostic markers. These discoveries not only deepen our understanding of UCEC but also open up new avenues for the development of innovative immunotherapy approaches in the treatment of this disease.

## Data Availability

The raw data supporting the conclusions of this article will be made available by the authors, without undue reservation.

## Supplementary material

Supplementary Figure 1 SLC2A1 and MPST gene alterations are not associated with the survival outcomes in UCEC. **(A, B)** OncoPrint visual summary of the alterations in the SLC2A1 and MPAT gene. **(C, D)** Kaplan–Meier survival curves show the overall survival rates of UCEC patients with or without SLC2A1 and MPST gene alterations.
