# Supplementary figures and images for: SLC2A1 and MPST as diagnostic and prognostic biomarkers of potential endometrial cancer
Source: Front Immunol. 2025 Jul 17;16:1575916. doi: 10.3389/fimmu.2025.1575916 (PMC12310493; doi:10.3389/fimmu.2025.1575916)

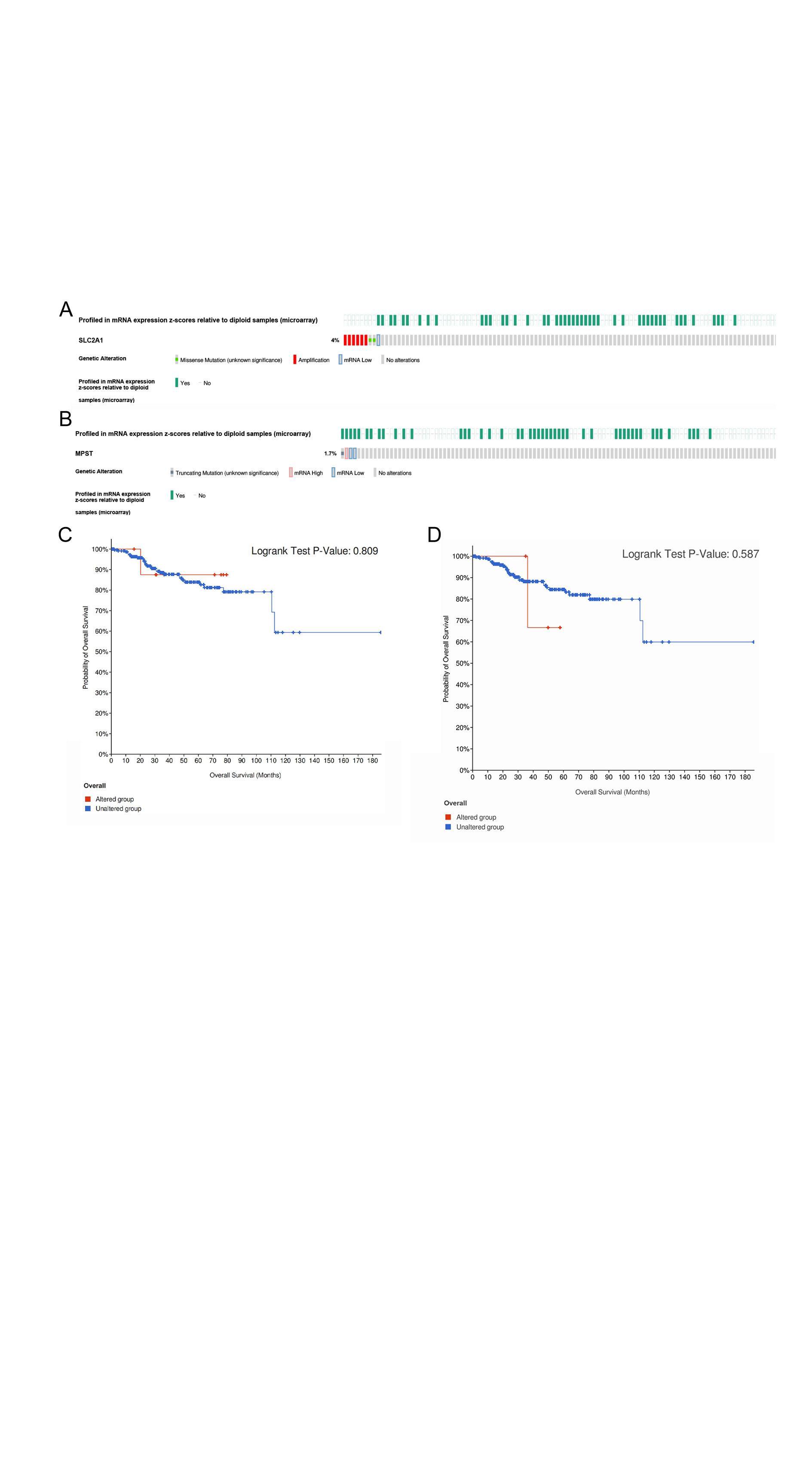

Supplement: Supplementary Figure 1 — SLC2A1 and MPST gene alterations are not associated with the survival outcomes in UCEC. (A, B) OncoPrint visual summary of the alterations in the SLC2A1 and MPAT gene. (C, D) Kaplan–Meier survival curves show the overall survival rates of UCEC patients with or without SLC2A1 and MPST gene alterations. [file Image1.tif]
